# Hereditary cataract in the Bengal cat in Poland

**DOI:** 10.1186/s12917-020-02517-x

**Published:** 2020-08-14

**Authors:** Natalia Kucharczyk, Anna Cislo-Pakuluk, Pawel Stefanowicz, Peter Bedford

**Affiliations:** 1 Przychodnia Weterynaryjna Viva, ul. Strachocinska 143, 51-518 Wroclaw, Poland; 2 Przychodnia Weterynaryjna Retina, ul. Szyllinga 3, 30-433 Krakow, Poland; 3 Royal Veterinary College Hatfield, Herts, UK

**Keywords:** Bengal cat, Congenital, Early onset, Cataract, Nuclear, Inheritance

## Abstract

**Background:**

This paper reports the significant prevalence of a presumed hereditary cataract in the Bengal cat breed in Poland. The nuclear part of the lens is affected and previous reports from Sweden and France for this type of feline cataract suggest that a recessive mode of inheritance is probably involved.

**Results:**

Presumed congenital or neonatal cataract involving the posterior nuclear part of each lens was initially diagnosed in a 12 month old male Bengal cat. As both parents and a sibling were also affected with cataract, a group of 18 related and 11 non-related cats was then subsequently examined. Eight related cats and one non-related cat were found to be similarly affected. A breed survey was then completed using an additional five centres across Poland and a further 190 related cats were examined. A total of 223 cats have been involved in this study, with 75 (33%) being affected with several types of cataract and 67 (30%) being specifically affected with the same or similar nuclear lesions. Eight cats (3.6%) presented with other cataract types and a prominence of the posterior lens suture lines was recorded in 65 cats unaffected with cataract (29%). There were no demonstrable vision problems. Neither age nor coat colour was significantly associated with the nuclear cataract, but the nuclear cataract group had a higher proportion of females than the unaffected group. Pedigree analysis has indicated probable inheritance as a recessive trait.

**Conclusions:**

These findings suggest that a presumably inherited nuclear cataract is present in the Bengal cat breed in Poland. It is considered to be either congenital or of very early onset, probably being inherited as a recessive trait. Although the lesion has no noticeable effect on vision, breeders in Poland and worldwide should be aware of the disease and clinical examination of young breeding stock prior to reproduction is advisable.

## Background

The Bengal cat breed found its origin in the USA as the result of crossing the Asian Leopard Cat (*Prionailurus bengalensis*) with a domestic cat breed (Felis Sylvestris catus), the purpose being to combine its unique physical appearance with the amenable character of a domesticated breed. The Bengal cat is now generally recognised as a very popular feline breed worldwide and it is enjoying its relatively recent introduction into Poland, with 4475 Bengal registrations being recorded in 2018 by the Polish Federation Felis Polonia (FPL).

A number of genetically determined ocular diseases have been recognized in several feline breeds, the most common of which are the lysosomal storage diseases, retinal dysplasia, progressive retinal atrophy and glaucoma [1–6]. Hereditary cataract would appear to be rare in cats, most reports describing mainly posterior nuclear and cortical lesions thought to be congenital or early onset in origin. Several breeds are involved including the Birman, the British Shorthair, the Himalayan, the Persian and the Russian Blue [7–12]. Cataract secondary to uveitis, trauma, neoplasia, nutritional deficiency or metabolic disease is more commonplace in this species [13–21]. A congenital cataract has also been noted in the feline Chediak-Higashi syndrome [22].

There are few studies relating to possible hereditary ocular disease in Bengal cats, but an early onset autosomal recessive retinal degeneration has been described [5] and recently nuclear and posterior polar subcapsular cataracts have been recorded in France [23].

## Results

The initial cataract diagnosis was made in a 12 month old entire pet male Bengal cat in the city of Wroclaw. The owner had only recently noticed a slight haziness in both eyes, particularly in low levels of illumination. Possible vision impairment was also suggested, but at clinical examination the performance in a maze test was adjudged to be normal. The menace response and the pupillary and dazzle reflexes were normal. A diagnosis of bilateral nuclear cataract with posterior cortical opacities was made and both fundi were seen to be normal. As both parents and a solitary male sibling were present in the same household these were then examined: both parents were found to be affected with nuclear cataract with minimal posterior cortical involvement and the sibling with minimal anterior and posterior cortical cataract. No other ocular defects were found in these four cats.

A group of 18 cats related to the same family together with 11 cats not related to the original case were then examined in a commercial cattery in the same area. Within the related group eight of the 18 cats (44%) were found to be affected with nuclear cataract and no non-nuclear opacities were seen. In the non-related cats only one (9%) presented with nuclear cataract and one non-nuclear anterior cortical cataract was found in one other cat (9%). Nineteen cats were clear for any type of cataract, but variable degrees of posterior suture line prominence was noted in 2 related and 3 non-related cats (17%).

The survey was then extended to involve another 190 cats that were related to the original case. It was completed in five additional centres in different parts of the country: 55 (29%) cats within this group were affected with nuclear cataract and six (3%) had non-nuclear cataract. Posterior suture line prominence was again recorded in 60 of the 129 unaffected cats in this group (32%).

Together with the four original cats a total of 223 cats have been examined in this study, 103 female and 120 male (46:54) with a mean age of 20 months (standard deviation (+/− 24) months) at the time of the examination. In total, cataract was diagnosed in 75 cats (34%), with 67 (30%) being nuclear and eight (4%) being non-nuclear cataracts. One hundred forty-eight cats (66%) were unaffected with cataract and the posterior suture line prominence was visible in 65 cats (29%). The sample was divided into 4 groups and Table 1 presents the information about ages and sex within each group.

**Table 1:** Age and sex of the 223 cats

	**Nuclear Cataract **(NC; *N* = 67)	**Other Cataract **(OC; *N* = 8)	**Suture Line Prominence Only** (SL; *N* = 65)	**Non-affected** (NA; *N* = 83)	**Between-Group Differences**
**Mean age** (in months)	23 (±26)	43 (±42)	8 (±8)	25 (±25)	SL < NA SL < NC
**Sex** (female / male)	32 / 35 (48:52)	5 / 3 (63:37)	35 / 30 (54:46)	31 / 52 (37:63)	% Females: SL > NA

Independent-samples *t*-tests showed that the suture line prominence group had a significantly younger age at examination than the unaffected group (*t*(146) = 5,4, *p* < 0.001) and the nuclear cataract group (*t* (130) = 4.56, *p* < 0.001), with no significant difference in age between the nuclear cataract group and the unaffected group. Chi-square tests of independence showed that there was a near-significantly higher proportion of females in the nuclear cataract group (*X*^2^ (2, *N* = 150) = 3.34, *p* = .07), and a significantly higher proportion of females in the suture line prominence only group (X2 (2, *N* = 148) =4.02, *p* = .045) than in the non-affected group. No other age and sex differences were found between the four groups. Analysis involving the other cataract group was not carried out because of the small sample size.

Although cataract was diagnosed in 75 cats, vision impairment had only been suspected in one individual, but this was not confirmed at the time of clinical examination. Vision problems were not reported in any other cats.

Details of the clinical findings for the affected cats are recorded in Table 2, with cataract involving nuclear material present in 67 of the 75 affected cats (89%) and 30% of the entire sample.

**Table 2:** The 12 types of cataract recorded in the Bengal Cat survey

**Nuclear cataract only**				
**Cataract Type**		**MeanAge (in months)**	**Sex (female/male)**	**Additional Ocular Findings**
**Focal nuclear**	16 (24%)	24 (+/− 26)	9F/7M (56:44%)	
**Total nuclear**	8 (12%)	19 (+/− 19)	2F/ 6 M (25:75%)	1 microphthalmia with 1xMRD lesion and 1xPPM strand
**Nuclear/perinuclear opacities**	10 (15%)	27 (+/−25)	5F/5M (50:50%)	
**Focal/posterior cortical opacities**	6 (9%)	14 (+/−15)	2F/ 4 M (33:67%)	
**Focal/posterior suture line opacities**	15 (22%)	11 (+/−15)	6F/ 9 M (40:60%)	bilateral MRD
**Focal/anterior suture line opacities**	4 (6%)	31 (+/−20)	4F/0 M (100:0%)	
**Focal/dust-like opacities**	8 (12%)	40 (+/−45)	4F/ 4 M (50:50%)	retinal scar, right eye
**Total Nuclear Cataract**	67	23 (2–138)	32F/35M (48:52%)	
**Other cataract types**				
**Anterior suture line opacities**	1 (13%)	3 (+/−0)	1F/0 M (100:0%)	
**Anterior cortical opacities**	1 (13%)	22 (+/−0)	0F/1M (0:100%)	
**Posterior cortical opacities**	3 (38%)	66 (+/− 58)	3 F/0 M (100:0%)	
**Both anterior and posterior cortical opacities**	2 (25%)	41 (+/− 41)	0F/2M (0:100%)	
**Pan-cortical pulverulence**	1 (13%)	39	1F/0 M (100:0%)	
**Total Other Cataract**	8	43 (+/−42)	5F/3M (63:37%)	

*F* Female, *M* Male, *PPM* Persistent pupillary membrane, *MRD* Multifocal retinal dysplasia

**Fig. 1:**
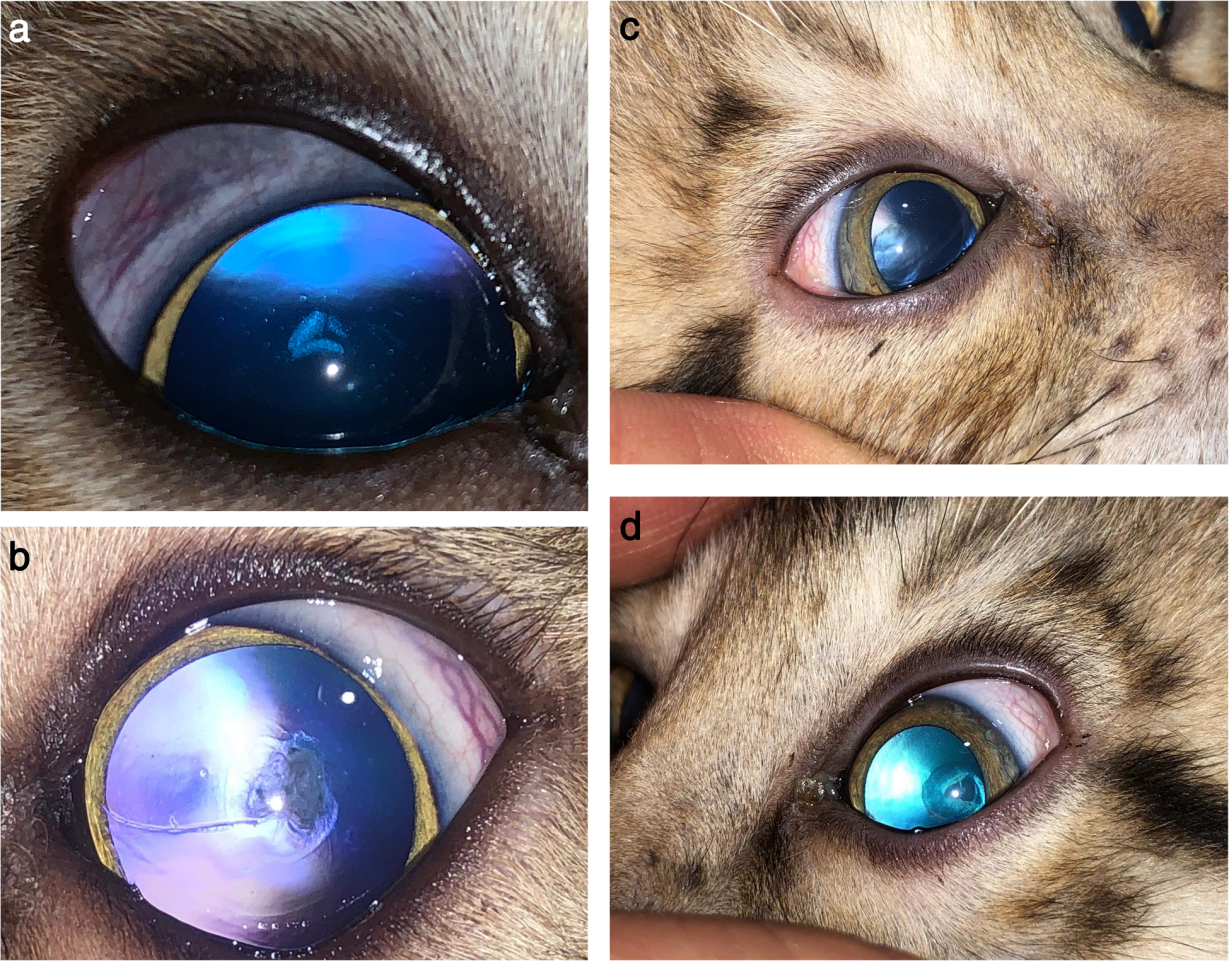
**a**. Focal nuclear cataract seen as a triangular opacity at the junction of the posterior nucleus and anterior cortex. Right eye. **b**. Focal nuclear cataract seen as a large triangular opacity in the posterior part of the nucleus. Left eye. **c**. Total nuclear cataract. Right eye. **d** Nuclear and perinuclear cataract. Left eye

**Fig. 2:**
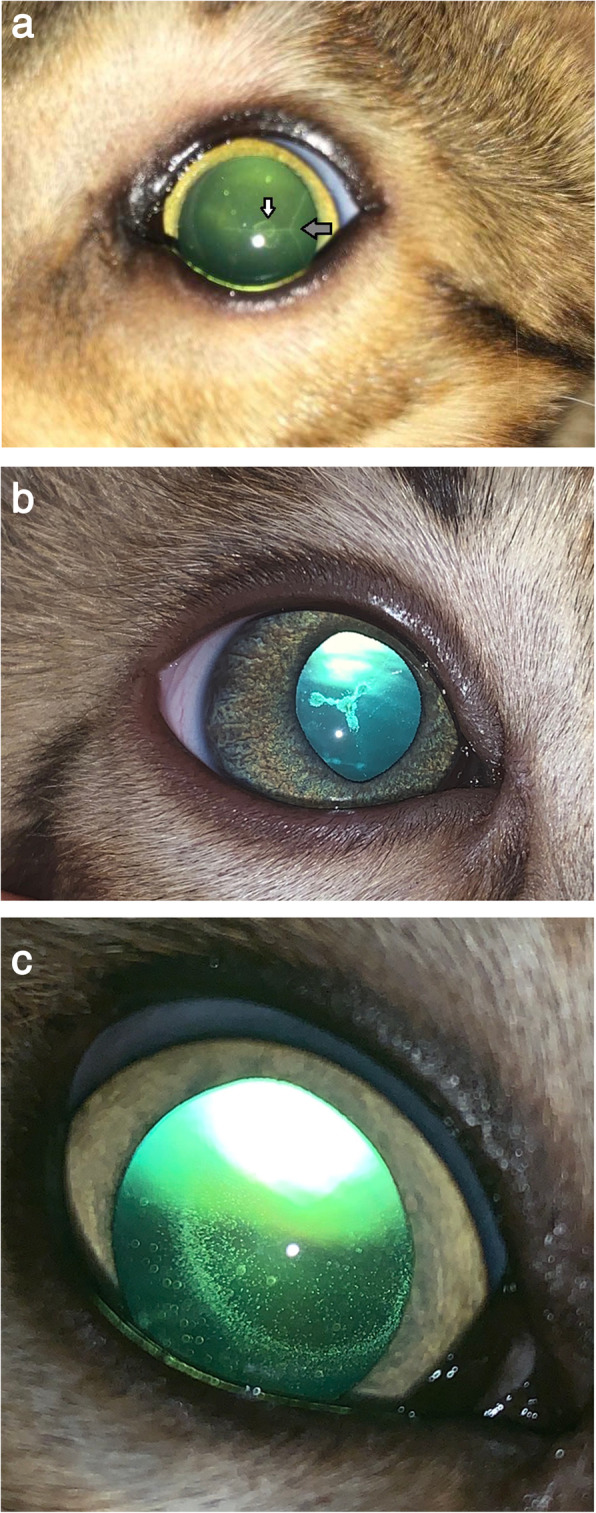
**a**. Nuclear cataract (white arrow) with posterior suture line cataract. (grey arrow). Left eye. **b**. Anterior suture line cataract. Right eye. **c**. Pulverulent cataract formation. Right eye

Seven types of nuclear cataract could be distinguished: 16 focal, eight total, 10 nuclear with perinuclear opacification, six focal with posterior cortical opacities, 15 focal with posterior suture line opacities, four focal with anterior nuclear suture line opacities and eight focal with dust like opacities. The focal cataracts were small triangular or Y-shaped opacities at the junction of the posterior nucleus and the anterior part of the posterior lens cortex (Fig. 1a and b). In the eight cats with total nuclear involvement the whole of what would have been foetal and embryonic lens material was opaque (Fig. 1c). The additional perinuclear involvement seen in the 10 cats with focal nuclear cataract was cortical opacity adjacent to and around the posterior part of the nucleus (Fig. 1d). The anterior nuclear suture lines seen in four cats with focal nuclear cataract were seen only with the slit lamp biomicroscope and in eight cats the nuclear opacities had a dust-like appearance. In 15 cats the focal cataract was seen with well-defined posterior suture lines (Fig. 2a).In the remaining eight cats there were five different presentations with no nuclear involvement: one had anterior suture line opacities (Fig. 2b), one had small anterior cortical opacities, three had small posterior cortical opacities, two had both anterior and posterior cortical opacities and one had pan-cortical pulverulence (Fig. 2c).

The additional ophthalmic findings in three cats with nuclear cataract included one cat with bilateral retinal folding indicative of retinal dysplasia, one cat with unilateral post inflammatory retinal scarring and one cat with microphthalmia with a unilateral MRD lesion and a single iris to iris strand of persistent pupillary membrane. Apart from one two year old unaffected male in which there was patchy bilateral tapetal hyperreflectivity there was no other clinical evidence of the early onset retinal degeneration which has been recorded in this breed [5]. The aetiology of the tapetal hyperreflectivity in this cat was undetermined and the CEP290 rdAc mutation test was not completed at the owner’s request.

There are many variations in coat colour in this breed (FPL). Eight were seen in this study, the most common being a brown or black spotted tabby colour (*N* = 184;83% of the total sample). The seven other coat colours comprised 39 cats (17% of the total sample; see Table 2 for details).

For the brown/black spotted cats (*N* = 184), 29% had a nuclear cataract,3% had other cataract types and 28% had suture lines only. For cats of the seven other coat colours (*N* = 39), 36% had a nuclear cataract, 5% had other cataract types and 33% had suture lines only. A chi-square test of independence showed that there was no significant association between coat colour grouping and the type of cataract (*X*^2^ (3, *N* = 223) = 1.1, *p* = .95).

The details are to be found in Table 3.

**Table 3:** Coat colours and cataract status frequencies

**Coat Colour**	**Official Coat number**	**All Cats**	**Nuclear Cataract Only**	**Other Cataract Types**	**Suture lines only**	**Unaffected Cats**
**Brown/black spotted**	n24	184	53 (29%)	6 (3%)	52 (28%)	73 (40%)
**TOTAL non-Brown/black spotted**	7 types	39	14 (36%)	2 (5%)	13 (33%)	10 (26%)
**Snow spotted**	n24 33	8	4 (50%)	1 (12.5%)	1 (12.5%)	2 (25%)
**Brown/black marbled**	n22	11	4 (36%)	______	4 (36%)	3 (27.2%)
**Seal mink spotted**	n24 32	11	3 (27%)	1 (9%)	4 (36%)	3 (27%)
**Seal mink marbled**	n22 32	1	1 (100%)	______	______	________
**Black silver spotted**	xn 24	5	2 (40%)	______	1 (20%)	2 (40%)
**Seal sepia spotted**	n24 31	2	–	–	2 (100%)	________
**Blue spotted**	______	1	______	______	1 (100%)	________
**Total Sample**	8	223	67 (30%)	8 (4%)	65 (29%)	83 (37%)

**Fig. 3:**
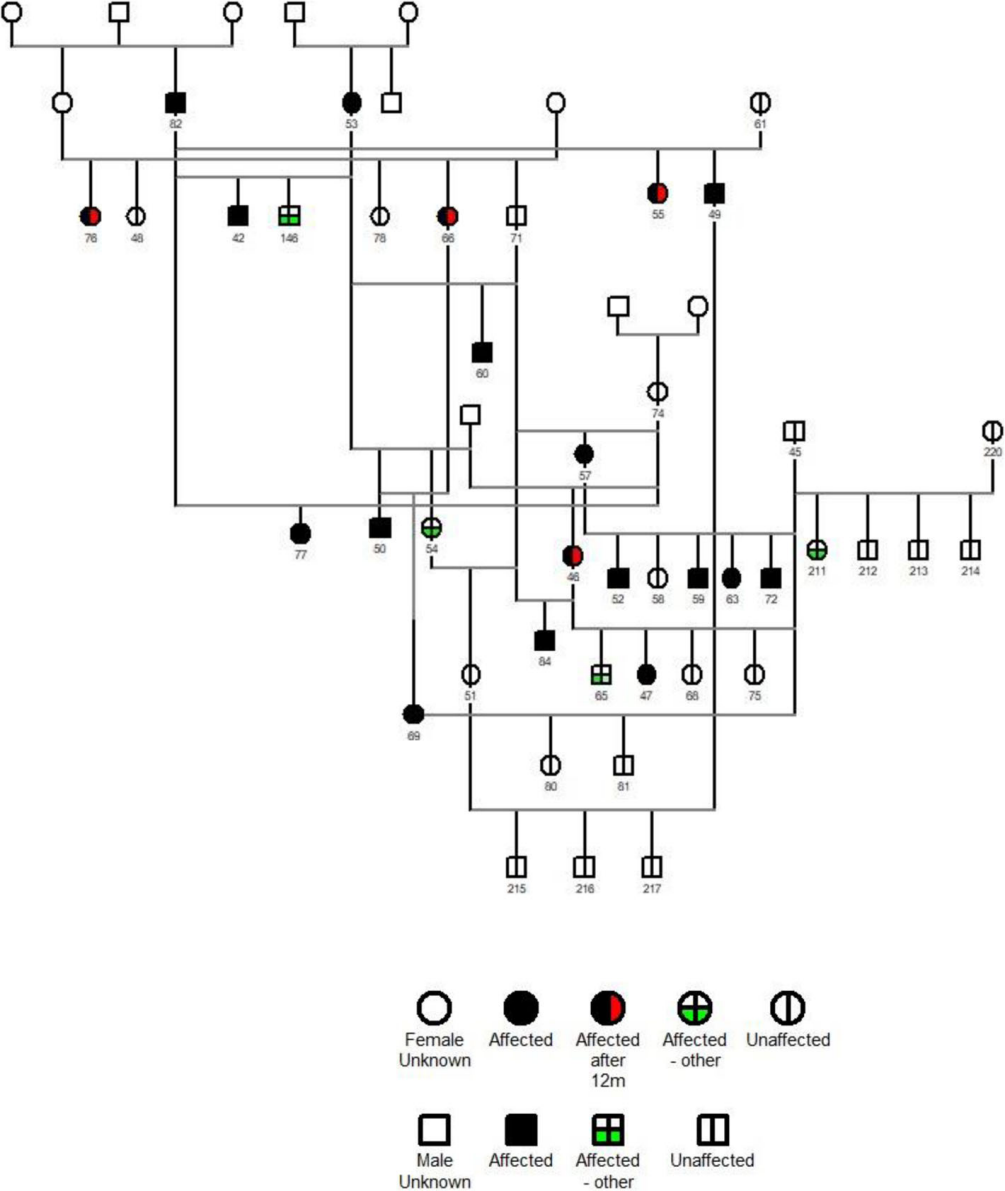
The pedigree of the first Bengal cat, number 42. The square symbols are males, the circular symbols are females, the affected cats with nuclear cataract, the half blank symbols are the cats with other cataracts and the divided symbols are unaffected cats. NB. cat 235 developed a pulverulent cataract after 12 months and the horizontal half blank symbol has been used to differentiate it from the other cats with non-nuclear cataract

**Fig. 4:**
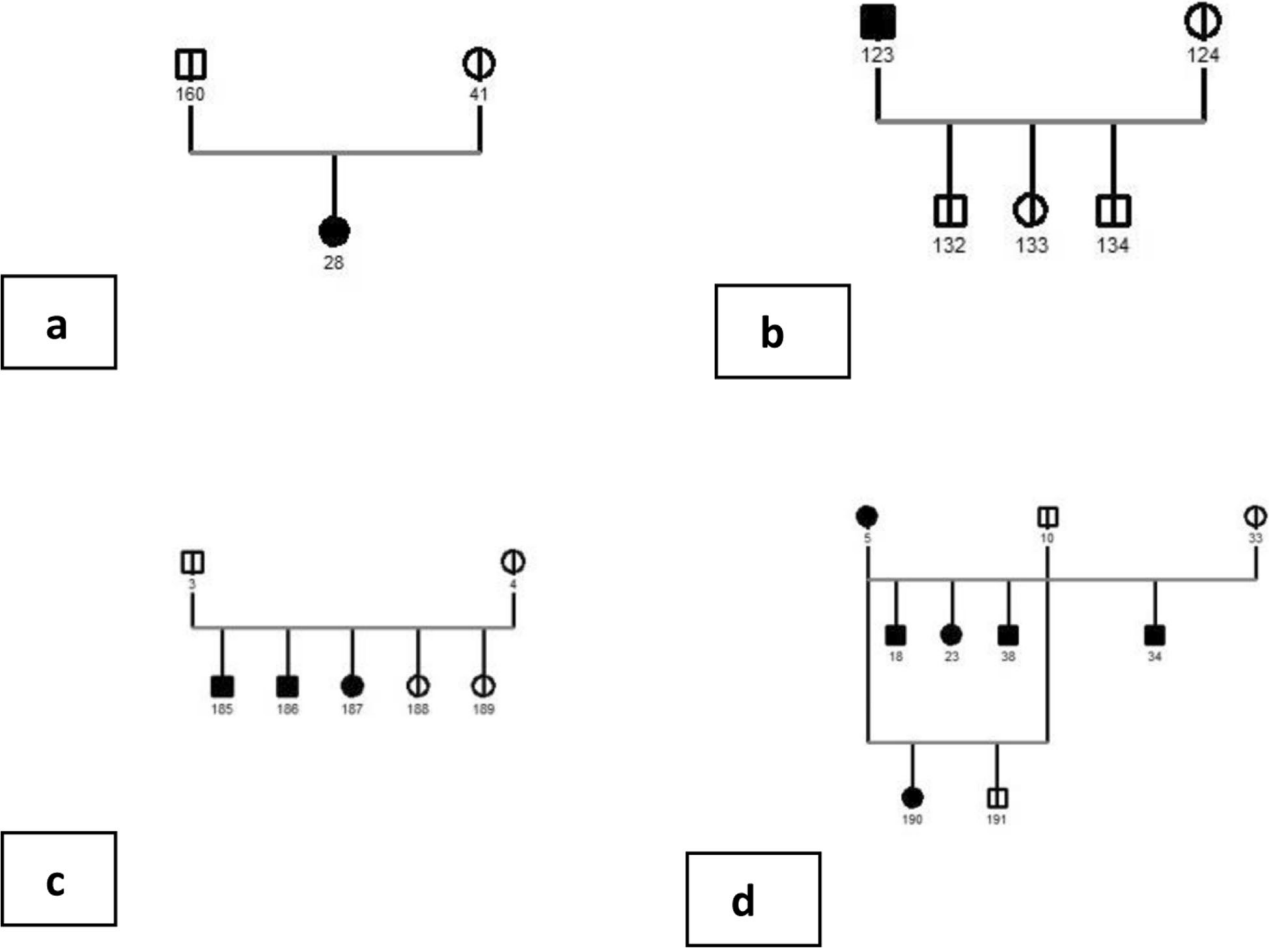
Four parts of the GENO PRO generated pedigree**: a**. two clear parents producing an affected cat. **b**. an affected /clear mating producing 3 clear offspring. **c**. a clear to clear mating producing clear and affected offspring. **d**. two affected/ clear matings producing affected and clear offspring

A GENO PRO software generated pedigree chart of the 223 cats was produced, but data could not be completed fully for all the cats: some lines could not be followed, but the main deficiency was that whole litters were not presented for examination. The analysis of the data suggests that an autosomal recessive pattern of inheritance is possible (Figs. 3 and 4), but without the accuracy of test mating, a dominant trait with incomplete penetrance cannot be ruled out. Figure 3 is part of the composite pedigree that details matings within the family related to the first affected cat seen (42). Cats 53 and 82 are the parents and 146 is the male sibling with anterior and posterior cortical cataract. Some of the matings in Fig. 3 that could suggest a recessive trait are the following: in a mating with clear male 45, affected cat 46 produced two clear cats (68 and 75), one affected cat with nuclear cataract (47) and one affected cat with non-nuclear cataract (65); in a mating with the affected cat 57, cat 45 produced a litter of four affected cats (52, 59, 63 and 72) with one unaffected cat (58); affected cat 69, produced by affected parents 66 and 50 mated with the unaffected cat 45 produced two unaffected cats (80 and 81); affected cat 46 was produced from one clear (74) and one unknown parent; cat 51 had both parents clear for nuclear cataract (54 and 71) and affected cat 49 mated with unaffected cat 51 produced three clear cats (215,216 and 217). The mating of affected cats 84 and 46 which resulted in two clear offspring (68 and 75), one affected with nuclear cataract (47) and one affected with non-nuclear cataract (65) is open to speculation.

In Fig. 4 four other parts of the GENO- PRO generated pedigree chart could demonstrate a possible recessive trait: in a first mating, affected cat 5 whose dam was unaffected was mated with cat 10 to produce three affected cats (18, 23 and 38) and in a repeat mating produced one affected female (190) and one unaffected male (191) (4d); when cat 10 was used again with an unaffected female (33) affected cat 34 was the result (4d); cat 123 mated with cat 124 produced three unaffected offspring (4b); the mating of two unaffected cats (160 and 41) produced one unaffected male (28) (4a) and as the result of the mating between unaffected cat 3 and unaffected cat 4 there were three affected (185, 186 and 187) and two unaffected cats (188 and 189) (4c).

In the cohort of 50 cats comprising 20 affected cats with nuclear cataract and 30 unaffected cats which was re-examined 12 months later, five of the unaffected cats had developed focal nuclear cataract and two were exhibiting nuclear pulverulence. Only one cat demonstrated a slight change in its nuclear cataract status in that the pulverulence was more defined. Two other cats with nuclear cataract had developed additional changes, one with a new punctate opacity in the anterior cortex close to the nucleus and the other an increased density in a posterior cortical opacity.

## Discussion

Cataract is the term applied to any opacity of the lens and /or its capsule. Its overall incidence in the feline species is relatively low, occurring usually as a secondary feature to other ocular or systemic diseases. In any species congenital cataract is present at birth, the opacities involving only the embryonic and foetal nuclear lens material [24]. In the canine species a congenital non-progressive nuclear cataract has been described as inherited in the Miniature Schnauzer and nuclear cataract has been recorded in other breeds including the Welsh Springer Spaniel [25], but there are few reports of this type of cataract occurring in the feline species. The significant prevalence of cataract involving the nuclear part of the lens reported here strongly indicates that this type of cataract is inherited in Bengal cats bred in Poland. In other feline breeds where cataract involving nuclear material has been reported the term congenital has been used, but elsewhere it has been suggested that such opacities may either be congenital or early onset in development [12, 26, 27]. In our study involving 67 Bengal cats with nuclear cataract there were 10 kittens aged under 5 months, confirming a very early development of the cataract whilst not denying a possible congenital origin. Neonatal kittens were not available at the time of this study, but even the earliest possible ophthalmic examination of kittens as young as 6 weeks of age might not provide the answer to this dilemma. In the Russian Blue study the youngest individual was 3 months of age. For the French Bengal cat survey the youngest cat in the observational study part of the programme was 9 months of age and in the referral group of 12 cats there were four three month old kittens [12, 26]. Older cats were involved in both parts of the study but the later ages at which the cats were examined do not indicate the age at which the cataracts may have developed. Whilst not denying a true congenital origin, both studies could also indicate that the cataract develops early in life. Similarly, most of the young unaffected Polish Bengal cats did not subsequently develop cataract, but in the subset of the 30 initially unaffected cats re-examined 12 months later, seven cats had developed lens opacities. Five of the seven presented with focal nuclear cataract and two with dust- like nuclear opacities. Two of the cats with nuclear cataract were less than 6 months of age when first seen, but the other five cats, three with nuclear cataract and two with the nuclear dusting, were above 2 years of age at first examination. These findings clearly demonstrate that there can be the occasional later development of what is generally considered to be congenital or very early onset disease.

The overall clinical picture is similar to that which has been described for the Russian Blue breed in Sweden and approximately the same as that for the Bengal cat in France [12, 23]. In the former report involving 66 cats with a cataract prevalence of 33%, triangular, Y-shaped or circular opacities were commonly present at the juncture of the posterior part of the nucleus and the anterior part of the posterior cortex. More extended opacities involved the whole nucleus, parts of the anterior or posterior cortex and the entire cortex. In the Polish Bengal cats focal nuclear and focal nuclear with posterior suture line opacities accounted for 46% of the cataract seen, with only 11% presenting with total nuclear cataract. In the French study involving a reported total of 63 Bengal cats, 14 nuclear and 10 posterior polar subcapsular cataracts were recorded. Both types were described as mainly bilateral, symmetrical and apparently non- progressive. The nuclear opacities were classified as focal, perinuclear, posterior nuclear and total nuclear, this clinical picture being approximately the same as that seen in the Polish Bengal cats. Some variations in the extent of cataract involvement in the nuclear and perinuclear parts of the lens are to be expected as possible manifestations of the same disease. However the notable difference between the French and the Polish Bengal cat populations is the presence of a posterior polar subcapsular cataract in 16% of the affected French cats, an opacity not seen in the 75 Polish Bengal cats presenting with the 12 types of cataract recorded here. In the canine species posterior polar subcapsular cataract is inherited in several breeds, developing in young dogs as early as 8 months of age [25]. This type of cataract is usually of little or no clinical significance, normally remaining non-progressive throughout life. It is possible that in the French Bengal cat its reported presence indicates that a second type of possible inherited cataract is present in the lines found in the breed in that country [23]. Posterior polar subcapsular cataract as a primary defect has been recorded in Burmese and Himalayan cat breeds where the lesions can be more extensive than those reported in the French Bengal cat and progression to total cataract formation within 12 months is possible [9, 11].

A second notable difference between the French findings and this report is the prominence of the posterior suture lines in 29% of the Polish cats. Visualisation of suture lines is usually an occasional finding, the clinical significance of which is not known. As suture lines mark the interdigitation of lens fibres they may effect variation in the focal length of light, but their significance in possible lens pathology is uncertain. It is generally believed that cataract may develop along suture lines and in the French report the posterior polar subcapsular opacities identified in 10 cats were described as small dots mainly along the suture lines [23]. It is of interest to note that so many of the cats not affected with cataract in our study had suture line prominence without opacity formation in the posterior cortex. However posterior suture line opacities were seen in 15 cats with focal nuclear cataract and similar opacities were seen in another 4 cats with the same focal nuclear cataract.

Nuclear cataract is normally considered to be non-progressive and this study would greatly support that conclusion. At re-examination of the 50 cats conducted 12 months later there appeared to be no change in the nuclear cataract status of the 20 affected cats, bar one. In this cat the nuclear pulverulence originally diagnosed had become marginally more defined. The only other changes seen were the development of an additional punctate opacity in the anterior cortex close to the nucleus in another cat and a slight change in the density of a posterior cortical opacity in another.

The possible impairment of vision suspected only in one cat could not be confirmed by maze testing and apart from the cataract no other ocular defect was found. No further noticeable sight defect has subsequently been observed for this cat and sight problems were not reported in any other cats affected with nuclear cataract over the following 12 months; however maze testing was not possible throughout the whole study due to the limitations of the clinical facilities available. Similarly vision problems were not reported in the French study [23]. It may be concluded therefore that this cataract has no material effect on vision in the Polish Bengal cat and that the concern of owners can be further tempered with the fact that the lesion would appear to be normally non-progressive. Significantly, the larger nuclear and cortical opacities reported in Russian Blue cats [12] were considered to be responsible for vision impairment or blindness in 27% of the 22 affected cats.

Analysis of the French report may have suffered from the fact that the 12 cats in one group were all presented with previously diagnosed cataract, whilst the cataract status of the larger group of 51 cats was unknown prior to the study. Our study and the French report differ somewhat in that cats from all over Poland were examined, the results suggesting a widespread distribution of the cataract. 82% of the French cats were all from the Ilse de France region of France, with just 11 related cats from other regions.

In both the Swedish and the French reports the authors have concluded that the development of this nuclear cataract must involve an hereditary component, with the Russian Blue study suggesting an autosomal recessive mode of inheritance [12]. The pedigree analysis demonstrated that whilst matings between affected parents produced affected offspring, unaffected parents also produced affected offspring. However, due to the fact that the study involved a relatively small number of cats, the analysis could not rule out the possibility of an autosomal dominant mode of inheritance with incomplete penetrance. The pedigree data used in the French Bengal cat report are compatible with an autosomal recessive pattern, but the authors conclude that further studies are necessary. It has been suggested that the focal and perinuclear cataracts seen in related Persian cats may be due to an autosomal dominant pattern of inheritance [27]. Gender is not considered to be a factor in either the Russian Blue or the French Bengal cat reports and both coat colour and gender are not dictating factors in our study. There was no association between the presence of nuclear cataract and coat colour, but a higher proportion of female cats had prominent posterior suture lines and there was a near significantly higher proportion of female cats in the nuclear cataract group than in the unaffected group. Given that there could be possible inconsistencies particularly in early pedigree data and that whole litters could not be examined, our study tends to support a possible autosomal recessive trait for this disease. However without the benefit of specific test matings a dominant trait with incomplete penetrance cannot be ruled out.

Other ophthalmic conditions were found in only four cats: the microphthalmia did not seem to affect the cat’s behaviour detrimentally and both the retinal folding and the post inflammatory retinopathy had no noticeable effect on sight. The bilateral tapetal hyperreflectivity diagnosed in one cat was indistinguishable from the early onset autosomal recessive degeneration due to a CEP290 rdAc mutation which has been described for this breed [5]. Despite the extent of the degeneration defective vision was not demonstrable in this cat and there were no other similarly affected cats in the study.

## Conclusions

The significant prevalence of a congenital or early onset cataract which involves the nuclear part of the lens strongly indicates that this type of cataract is inherited in the Bengal cat in Poland. Together with similar findings in France, this further suggests that the cataract may be present in this popular breed of cat worldwide. Fortunately the lesion usually only involves the small central part of the lens and appears to remain non-progressive, such that there would appear to be little impact on vision. Breeders should be aware of the potential inherited nature of this disease and in the absence of extant clinical features, the clinical examination of all young stock prior to breeding is the only disease control measure possible, until DNA testing becomes available.

## Methods

Following the initial examination of four related cats, three of which had nuclear cataract, another group of 18 relatives and 11 unrelated cats was examined in the same Wroclav area. As 44% of the related cats was affected with nuclear cataract, a further survey involving another 190 related cats was undertaken. To assess the situation across the whole country 5 additional centres in the Rokietnica/Poznan, Bydgoszcz/Torun, Krakow and Konin districts were used and the survey was completed within a month. All the cats were volunteered for examination by interested breeders and owners and written permission was obtained for the publication of pedigrees and results for all 223 cats involved.

Examinations were conducted both in veterinary clinics and in suitable rooms in catteries and households. The examination technique used throughout included maze testing, the assessment of the pupillary and dazzle reflexes in normal room light, with the rest of the examination being completed in appropriately darkened premises. Biomicroscopic evaluation (Kowa SL-15 portable slit-lamp, Kowa) was conducted before and after tropicamide induced mydriasis (Tropicamidum WZF 0.5%, Polfa Warszawa). Fundus examination following mydriasis was completed using indirect ophthalmoscopy (Keeler Spectra Plus) utilizing a 30D condensing lens (Volk Aspheric). Where temperament permitted photographs of the several cataract types were taken using an Iphone X camera (Apple).

Age, sex and coat colours were recorded to demonstrate any possible relationship between these factors and the presence and type of cataract.

Pedigrees were provided by the breeders and some data for the pedigree analysis covered approximately 12 years of breeding. There is no official Bengal Cat association in Poland and no regulatory body for the verification of individual identification and pedigree details. Cheek swabs from all the affected cats and 80 non-affected cats have been submitted for ongoing DNA analysis by the Animal Health Trust.

A cohort of 50 cats comprising of 20 affected with nuclear cataract and 30 unaffected cats from the original 223 cats was re-examined 12 months later to look at the possibilities of later onset in unaffected cats and potential change in the cataract status of affected cats.

## Data Availability

The datasets used in the current study are available from the corresponding author on reasonable request.

## Abbreviations

F: Female
M: Male
PPM: Persistent pupillary membrane
MRD: Mulifocal retinal dystrophy
FPL: Polish Federation Felis Polonia
